# Investigating the Single Trial Detectability of Cognitive Face Processing by a Passive Brain-Computer Interface

**DOI:** 10.3389/fnrgo.2021.754472

**Published:** 2022-04-08

**Authors:** Rebecca Pham Xuan, Lena M. Andreessen, Thorsten O. Zander

**Affiliations:** ^1^Technical University Berlin, Naturalistic Driving Observation for Energetic Optimization and Accident Avoidance, Institute of Land and Sea Transport Systems, Berlin, Germany; ^2^Volksagen AG Group Innovation, Wolfsburg, Germany; ^3^Neuroadaptive Human-Computer Interaction, Brandenburg University of Technology Cottbus-Senftenberg, Cottbus, Germany

**Keywords:** face recognition, passive brain–computer interface (pBCI), single-trial classification, automated driving, human-computer interaction

## Abstract

An automated recognition of faces enables machines to visually identify a person and to gain access to non-verbal communication, including mimicry. Different approaches in lab settings or controlled realistic environments provided evidence that automated face detection and recognition can work in principle, although applications in complex real-world scenarios pose a different kind of problem that could not be solved yet. Specifically, in autonomous driving—it would be beneficial if the car could identify non-verbal communication of pedestrians or other drivers, as it is a common way of communication in daily traffic. Automated identification from observation whether pedestrians or other drivers communicate through subtle cues in mimicry is an unsolved problem so far, as intent and other cognitive factors are hard to derive from observation. In contrast, communicating persons usually have clear understanding whether they communicate or not, and such information is represented in their mindsets. This work investigates whether the mental processing of faces can be identified through means of a Passive Brain-Computer Interface (pBCI). This then could be used to support the cars' autonomous interpretation of facial mimicry of pedestrians to identify non-verbal communication. Furthermore, the attentive driver can be utilized as a sensor to improve the context awareness of the car in partly automated driving. This work presents a laboratory study in which a pBCI is calibrated to detect responses of the fusiform gyrus in the electroencephalogram (EEG), reflecting face recognition. Participants were shown pictures from three different categories: faces, abstracts, and houses evoking different responses used to calibrate the pBCI. The resulting classifier could distinguish responses to faces from that evoked by other stimuli with accuracy above 70%, in a single trial. Further analysis of the classification approach and the underlying data identified activation patterns in the EEG that corresponds to face recognition in the fusiform gyrus. The resulting pBCI approach is promising as it shows better-than-random accuracy and is based on relevant and intended brain responses. Future research has to investigate whether it can be transferred from the laboratory to the real world and how it can be implemented into artificial intelligences, as used in autonomous driving.

## Introduction

Face recognition is considered a highly important skill for humans. It also plays an important role in daily road traffic, especially in the transition period toward fully automated driving. Normally, drivers solve unclear situations with non-verbal communication (i.e., eye contact, mimicry). An autonomous car might have decided to execute a certain action but might leave other traffic participants confused in complex situations. This can be caused by the missing eye contact, which is an important interaction element between the driver and vulnerable road users (Alvarez et al., 2019; Bergea et al., 2022) and promotes calm interactions (Owens et al., 2018). If the car does not compensate for this missing communication between drivers or pedestrians, a potentially dangerous situation might be the result.

In this study, we investigated a potential solution to this problem in semiautomatic driving. Here, the driver is intended to be aware of the ongoing situational context the car is in and should identify automatically non-verbal communication attempts of other road users or pedestrians. The car could gain access to this situational interpretation of the driver's brain by means of Passive Brain-Computer Interfacing (Zander and Kothe, 2011). In that way, the brain of the driver would serve as a sensor for the car, interpreting the environment and filtering out relevant information of non-verbal communication that could not be derived otherwise. The detection of the face of a person that starts a non-verbal communication can then be transferred to the car leading to further analysis of the environment and a revision of the actions currently planned. As passive BCIs rely on implicit control (Zander et al., 2014), the driver does not need to be aware of this information transfer to the car.

The work here takes a first step toward the above-described concept. It investigates whether a pBCI can be calibrated to distinguish brain responses related to the identification of human faces from those representing the mental identification of other objects or information. Therefore, the pBCI is calibrated in the laboratory where the different responses were evoked in a controlled setup. The resulting data were evaluated regarding the accuracy the pBCIs had in distinguishing the different stimuli and regarding the cortical sources that contributed to the signal used.

Studies using MRI or fMRI have shown a significantly higher activity in a certain brain area when participants see faces in contrast compared to, e.g., houses or other objects (Kanwisher et al., 1997, 1999). This area, which is especially sensitive to face perception, is the fusiform gyrus. The fusiform gyrus is part of the temporal lobe in Brodmann area 37. Trans Cranial Technologies ldt. (2012) claim that face recognition activates a widespread network in the brain. This network includes the bilateral frontal area (BA 44, 45), occipital (BA 17, 18, and 19), the fusiform gyri (BA 37), and the right hippocampal formation. Lesions in this area are associated with different manifestations of visual agnosia, e.g., object or face agnosia (Trans Cranial Technologies ldt., 2012). In some studies, activities have also been recognized in the region of the middle temporal gyrus/superior temporal sulcus (STS) (Perrett et al., 1987; Kanwisher et al., 1997; Halgren et al., 1999). The STS responds stronger to faces or its features than to other complex visual stimuli (Jeffreys, 1989). A different area probably also contributing to face perception is the lateral inferior occipital gyrus (Sams et al., 1997). Bötzel et al. have shown the topographic display of face-evoked potentials. The results lead to the assumption that the face-evoked potential is very strong on electrode Cz as well as on the parietal hemispheres (Bötzel et al., 1995).

Different single module studies locate different types of brain activity when a face is perceived. This has opened a debate of whether face perception is mainly done by one module specialized to faces (Kanwisher et al., 1997) or distributed processing. In their work, Haxby et al. collected the outcomes of different studies and built a model of face perception areas in the brain (Haxby et al., 2000). The author's model demonstrates that face perception activates the core system (occipitotemporal visual extrastriate areas) as well as an extended system (neural systems whose functions play a role in extracting information from faces). The model has a more holistic approach since it considers that different areas are activated according to, e.g., the familiarity of a face, the shown emotion, or the features of the face.

Current ERP literature names two main components, which are connected to face-specific cortical activity: the N170 and the P1. Already, in the mid-1990s, it was described that the N170 is a bilateral potential at the occipital and posterior temporal electrodes (Bötzel et al., 1995), originating from the fusiform gyrus (Herrmann et al., 2004). It is face-sensitive as the response is larger with “face” than “no face” stimuli. It indicates not only head detection that is sensitive to the configurational analysis of whole faces (including the features within the face, as nose etc.) (Eimer, 2000). In fact, many studies of ERP correlate for face processing did not report or analyze anything about the P1 component (Herrmann et al., 2005). Most studies that investigate the P100 effect do report amplitude differences (Halit et al., 2000; Itier and Taylor, 2004; Herrmann et al., 2005), but few other studies did not confirm those findings (Rossion et al., 2003). Herrmann et al. (2005) supposed the different findings could be due to differences in low-level features between stimulus categories, such as luminance and contrast. As the P1 component is not fully understood yet, it should be used with caution until further investigations have solved the inconsistencies.

The existing knowledge in the field of face recognition allows taking a further step toward detecting facial recognition in a single trial with a pBCI. The main research question of this work aims at investigating the BCI-classifiability of face perception that is processed in the fusiform gyrus. This includes determining the single-trial classification accuracy, as well as the neuroscientific identification of features contributing to classification. The implementation of the experiment is based on the work of Deffke et al. (2007). As in the study made by Itier and Taylor (2002), eight electrodes were considered as important: the temporal-parietal sites TP9 and TP10, the posterior parietal sites P7 and P8, the occipito-parietal sites PO9 and PO10 as well as the occipital electrodes O1 and O2. A further important electrode is Cz as the “face-specific brain potential is most prominent here” (Herrmann et al., 2002). This goes along with the former mentioned findings by Bötzel et al. (1995).

## Materials and Methods

In this study, next to faces, two other types of stimuli were shown, and participants had to answer whether the shown stimulus was a face or not. Further details of the participants and the experiment are given below.

### Participants

Thirteen participants were recruited from the Human Factors Master's Program at the Technical University in Berlin, Germany. They were either paid by earning 30€ or by collecting points for taking part in experiments, which are mandatory for graduating in the Master's program mentioned above. Data of the two participants had to be discarded due to technical difficulties. Therefore, only the data of 11 participants were taken into account for the analysis. The participants were between 24 and 34 years old; three were female. All of them had normal or corrected-to-normal (three participants) vision, and none of them reported physical or mental illness for the time of the experiments. The Participants gave written consent to take part in the study.

### Experimental Setup

Sixty-four channels of EEG were recorded with two amplifier modules (BrainAmp32 DC) provided by the company Brain Products GmbH. Electrodes were placed according to the International 10–20 system, with the ground electrode placed at position AFz, while electrodes were referenced to FCz. In addition, the electrooculogram (EOG) was recorded. All electrodes had impedances lowered to 5 kΩ. The participants were placed in a comfortable chair with armrests with about 60–70-cm distance to the monitor, while lighting conditions were constant during the experiment.

**Figure 1:**
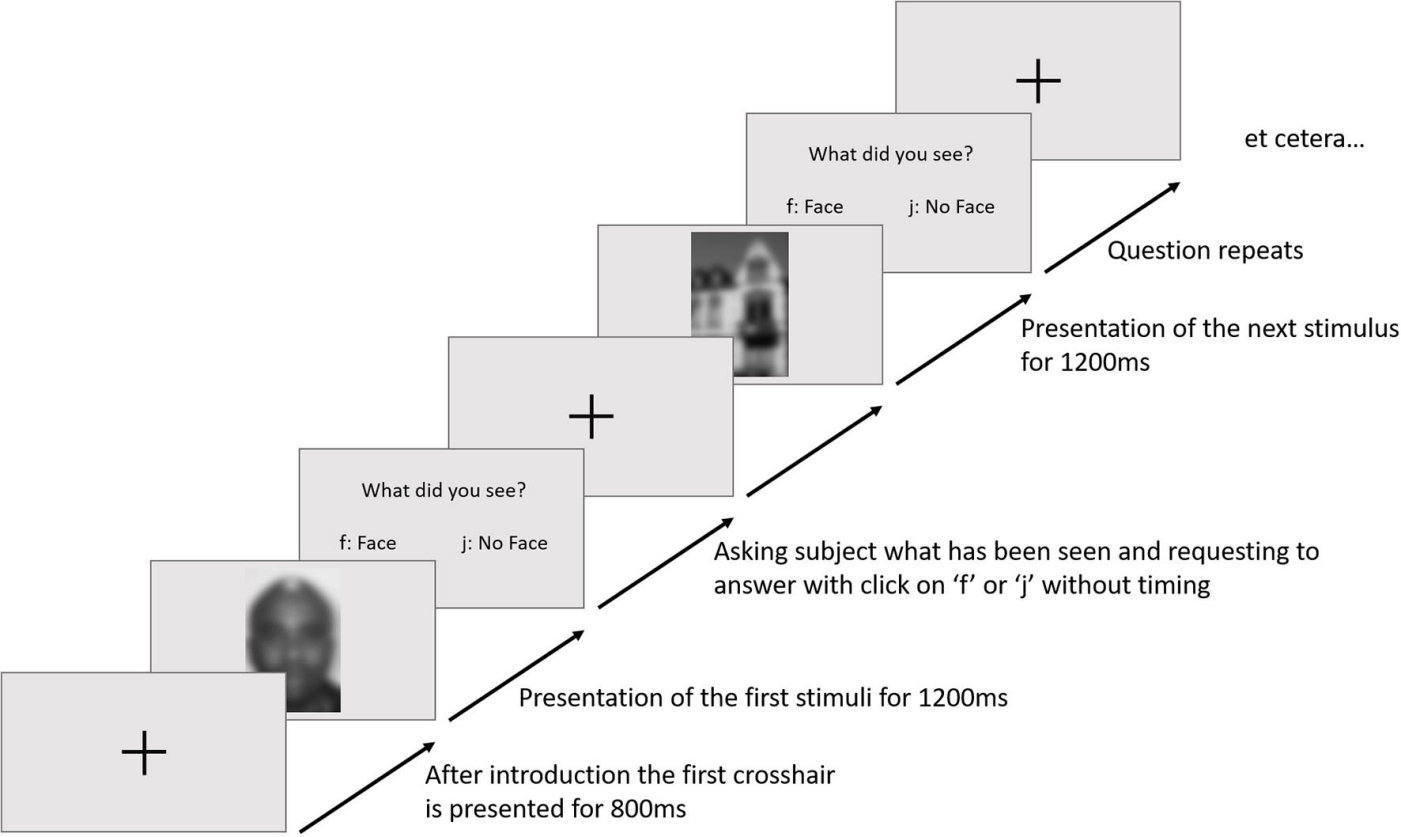
An example of a trial sequence. Blurred images for privacy.

The participants were welcomed and introduced to the experimental task. After being seated, the electrode cap was put on; gelled and EOG electrodes were fixed. At the beginning of the paradigm, the participants were told that the experiment was about the differences in recognizing a face and something else. Each trial started with a fixation cross (800 ms), followed by a stimulus (1,200 ms) in the center of the visual field. An example of the trial sequence is given in Figure 1. Afterwards, a question appeared whether a face had been seen. The answer was given by pressing a button with the index fingers of their two hands (as in the experimental paradigm of Collin et al., 2012).

Trials would repeat for the whole size of a block. In between each block, the participants had to take a mandatory break for 2 min, reminding them to not answer the question before the stimuli disappeared. The whole experiment consisted of 6 blocks each, having 90 trials, so 540 stimuli were shown overall. The stimuli were randomized and were marked with “face” for faces, “no face” for houses and “maybe face” for abstract images in the EEG. Depending on the participants' answers, a marker was sent (“responseFace,” “responseNoFace”). During the procedure, the participants got no kind of feedback on the validity of their answers.

### Stimuli

Perceiving face stimuli might evoke different brain responses next to that being specific to faces. These include the identification of a concrete object (e.g., the human head) and the processing of abstract information (information encoded in the facial features). To ensure that the pBCI was calibrated on brain responses specific to face recognition only, two different types of counter-stimuli were used: houses, representing concrete objects, and abstract pictures reassembling some familiarity with faces, but not showing any concrete, known shape. With that, the counter-stimuli evoke brain responses to object recognition and to the processing of abstract information, but none specific to face recognition. In that way, the pBCI can only rely on the specific brain responses evoked by faces when trying to discriminate the EEG activity evoked by face recognition from that evoked by perceiving houses or abstract shapes. All pictures were in greyscale and had the same format. The “face”-stimuli were downloaded from a database provided by UT Dallas. About 180 pictures from the Minear and Park database were used to create a database of normed faces, reflecting age and ethnic diversity. All pictures were from the forward-facing profile with a neutral expression (Kennedy et al., 2009). Half of the people in the displayed pictures were 18–49 years old; the other half, older. Distribution was equal between the genders. This sample was chosen intentionally to represent an ethnically diverse sample. The used stimuli representing obvious “no faces” were pictures of houses. The pictures were retrieved from real estate pages and displayed single family houses and apartment buildings. The stimuli in the category “maybe faces” have been selected off a database from the MIT Center for Biological and Computational Learning. The purpose of the pictures was to generate a dataset to train a Support Vector Machine classifier, which should detect frontal and near-frontal views of faces. In order to achieve high robustness for within-class variations (changes in illumination, background, etc.), stimuli containing faces in different processing stages were compared. Among those were the used stimuli, which were Haar wavelets generated by a single convolution mask originating from face pictures (Heisele et al., 2000). As those pictures were originally face pictures but then processed to unrecognizability, this stimuli set served as “maybe faces.”

## Data Analysis and Statistics

In order to analyze the data, MATLAB and the open source toolboxes EEGLAB and BCILAB were used. EEGLAB was used for pre-processing, analyzing, and visualization of ERPs (Delorme and Makeig, 2004). The toolbox BCILAB was used to extract and classify features (Kothe and Makeig, 2013). Pre-processing of the EEG data is based on “Makoto's pre-processing pipeline” provided by the Swartz Center for Computational Neuroscience at UCSD, USA (Makoto, 2016). Any command described was used in a default mode if not labeled otherwise. After minimal pre-processing, an Independent Component Analysis (ICA) was run on the data, and the resulting quality of the ICA was examined (Jung et al., 1998; Hyvärinen and Oja, 2000). Eye components could be identified and further considered in the analysis. The examination of the ERPs and the actual training and testing of a classifier, as well as the inspection of the cortical activity related to face recognition, are reported below. Statistics with a *p*-value < 0.05 were considered as being significant.

### ICA Analysis

The ICA decomposes minimally pre-processed EEG data into statistically independent time series, so called components. These components can be distinguished by their spatial projection pattern and their activity. Based on these features, ICs representing cortical activity can be distinguished from those reflecting artifact activities or cannot be clearly associated with either of those source types. Here, only cortical components were kept, while all others, like eye components, were discarded.

The EOG has a dedicated reference electrode and cannot be compared to EEG channels. Consequently, EOG channels had been discarded before ICA was applied. To identify components reflecting a high share of brain activity, several indicators were used. One aimed at the residual scalp map variance (RV), which had to be lower than 10% from the best-fitting equivalent dipole to be considered as a brain component (Delorme et al., 2012). Obvious time series artifacts were discarded by manually scrolling through the data. Therefore, a highpass filter (1 Hz) and a lowpass filter (200 Hz) were applied. We chose this rather high frequency of 200 Hz to include muscle artifacts in our analysis and to ensure a quick response in the passive BCI classification. One dataset was recorded with the AC power line fluctuations (50 Hz line noise and harmonics) being a very dominant artifact. Harmonics are signals with an integer multiple frequency of the original frequency. Therefore, in all datasets, 45–55 Hz, 95–105 Hz, and 145–155 Hz were removed by using the EEGLAB plugin CleanLine by Mullen (2012). Bad channels and datapoints were rejected by visual inspection of continuous data using obvious deviations from standard EEG signals as the criterion. This included low drifts and high-frequency epochs. Channels were rejected when more than 20% of their data were considered being artifactual. After the manual rejection, an automatic rejection of artifact channels using joint probability was performed using pop_rejchan(). It was restricted to not discard the channels in which the ERPs for face recognition are assumed.

The cortical source of each IC was estimated by the following procedure: The topography had to imply that the source is a clear dipole located in the cortex; the activity power spectrum had to show activity within alpha or beta band for a component to represent brain activity; the intertrial coherence had to show any form of phase-locked activity and residual variance of the source localization done by the EEGLAB plug-in tool DIPFIT by Robert Oostenveld and Arnaud Delorme had to be below 10%. Clusters of ICs were generated across subjects by grouping ICs with a similar dipole and activity pattern. Only those clusters containing independent components from at least five participants were considered representative.

### ERP Analysis

Noisy channels with visibly corrupted data were removed manually. An automatic rejection process computed the joint probability by kurtosis. Re-referencing was then used to convert the dataset to a common average reference. We decided to remove channel Oz from the data to reduce the rank according to the average referencing of the data for ICA processing. O1, Oz, and O2 are relatively close to one another, meaning interpolating Oz should still bring good results, and using an electrode from the middle should keep the symmetry.

Before the epochs are extracted, a bandpass filter from 2 to 30 Hz is applied. In the present data, only little of the expected ERP could be seen if not highpass filtered with 2 Hz, which is probably caused by the Readiness Potential. The epochs extracted had a length from 800 ms with a starting point 100 ms before the stimulus onset. At the baseline, 100 ms before the stimulus onset were subtracted.

### Classification

Features were extracted along the windowed means approach (as described in Blankertz et al., 2011). Data were resampled at 100 Hz and bandpass filtered in a range of 0.1–10 Hz. In each trial and for each channel, features were then extracted by obtaining the average of each of 6 consecutive 50 ms windows, starting at 200 ms after the stimulus onset. This resulted in a 6-x-64 dimensional feature vector for each trial.

Three classifiers were generated for each participant through applying regularized discriminant analyses (LDA) to each pair of stimuli types and optimized to differentiate the classes in a binary fashion. To estimate the validity of each classifier, accuracy rates were generated by repeating 10-fold randomized cross-validation (CV). This procedure was repeated three times, resulting in an averaged accuracy rate as a performance measure.

## Results

On average, 60.18 (27.37%) of the ICs met the criteria of RV < 10%. Further components were discarded according to the procedure described in? 3, resulting in an average of 15 components per subject, which could still be considered as brain components, while the rest was considered bearing no relevant information. Only those clusters containing independent components from at least five participants were considered representative; the resulting 11 scalp maps are depicted in Table 1.

**Table 1:** An average scalp map of computed clusters.

**Cluster (number of participants)**	**Scalp map**	**Cluster (number of participants)**	**Scalp map**	**Cluster (number of participants)**	**Scalp map**
3 (5 Ps)	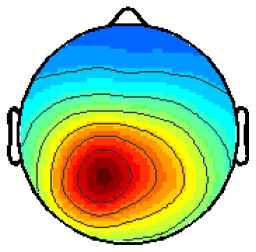	9 (9 Ps)	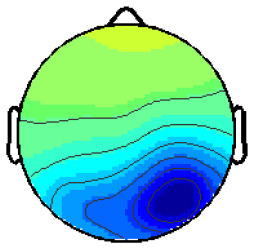	13 (9 Ps)	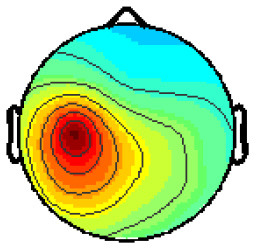
4 (8 Ps)	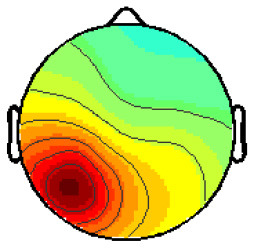	10 (6 Ps)	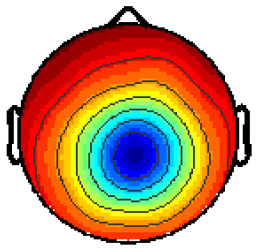	15 (7 Ps)	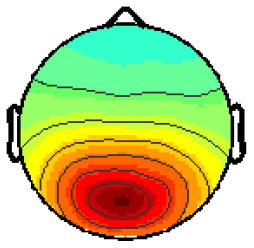
6 (6 Ps)	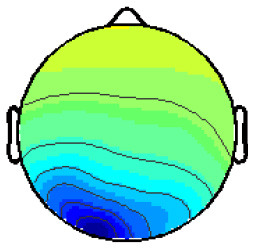	11 (6 Ps)	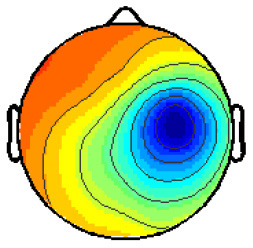	16 (6 Ps)	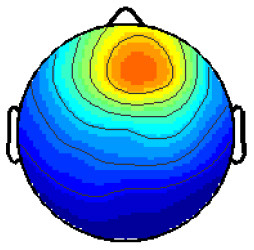
		12 (8 Ps)	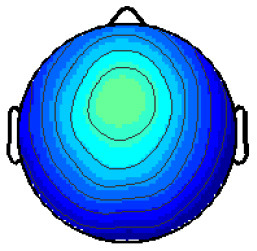	17 (5 Ps)	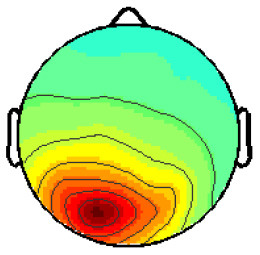

### ERP Results

#### EEG Data

**Figure 2:**
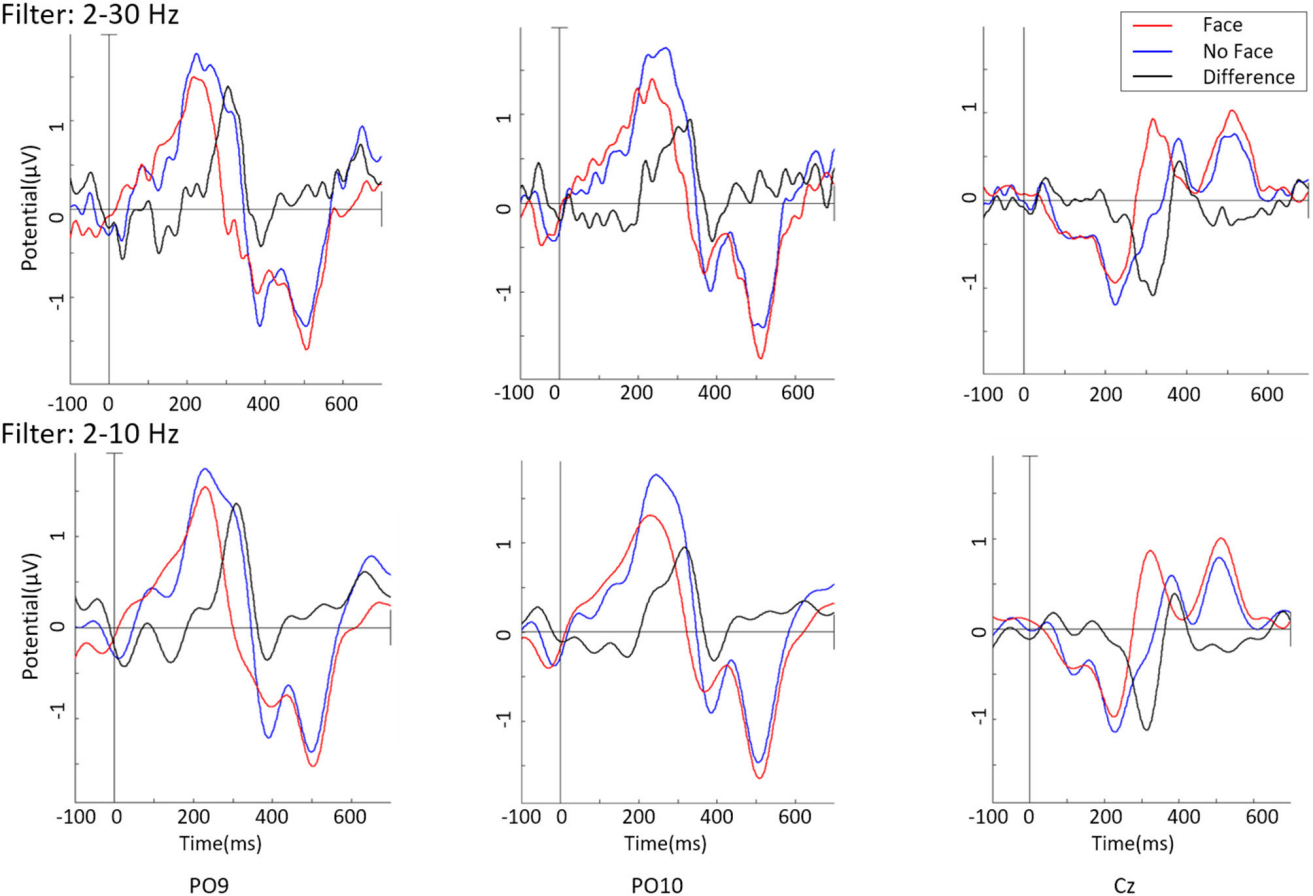
Grand average ERPs on the Channel PO9, PO10, and Cz with different bandpass filters.

Figure 2 (top) shows the grand averaged ERPs, on the channels PO9, PO10, and Cz, on data not containing eye components, bandpass filtered 2–30Hz. While the blue line describes the “face” response and the red line the “no face” response, the black line is the difference between these two. As noise at a higher frequency superimposed the data, a lowpass display filter of 10 Hz is applied (Figure 2, bottom). ERPs of face and no-face stimuli types show a similar morphology with minor differences in peak amplitudes. The difference of these ERPOs shows a clear peak around 200 ms.

#### ICA Data

One cluster (Cluster 6), to which six participants contributed, could be directly located in the fusiform gyrus, BA 18. The ERP resulting from this cluster is shown in Figure 2. The “face” stimuli shows a stronger N170 than the ERP for the “no face” stimuli. It has to be kept in mind that the ERP component polarity has to be inverted based on the scalp map polarity. The average negative peak of the face response for Cluster 6 is at the time of 286 ms. The “maybe face” stimuli evoke an even stronger potential than the “face” stimulus. This stays the same for the P1, for which the potential of “face” is clearly stronger than of “no face.” Before, at the N170, no obvious difference between “face” and “no face” could be seen.

**Figure 3:**
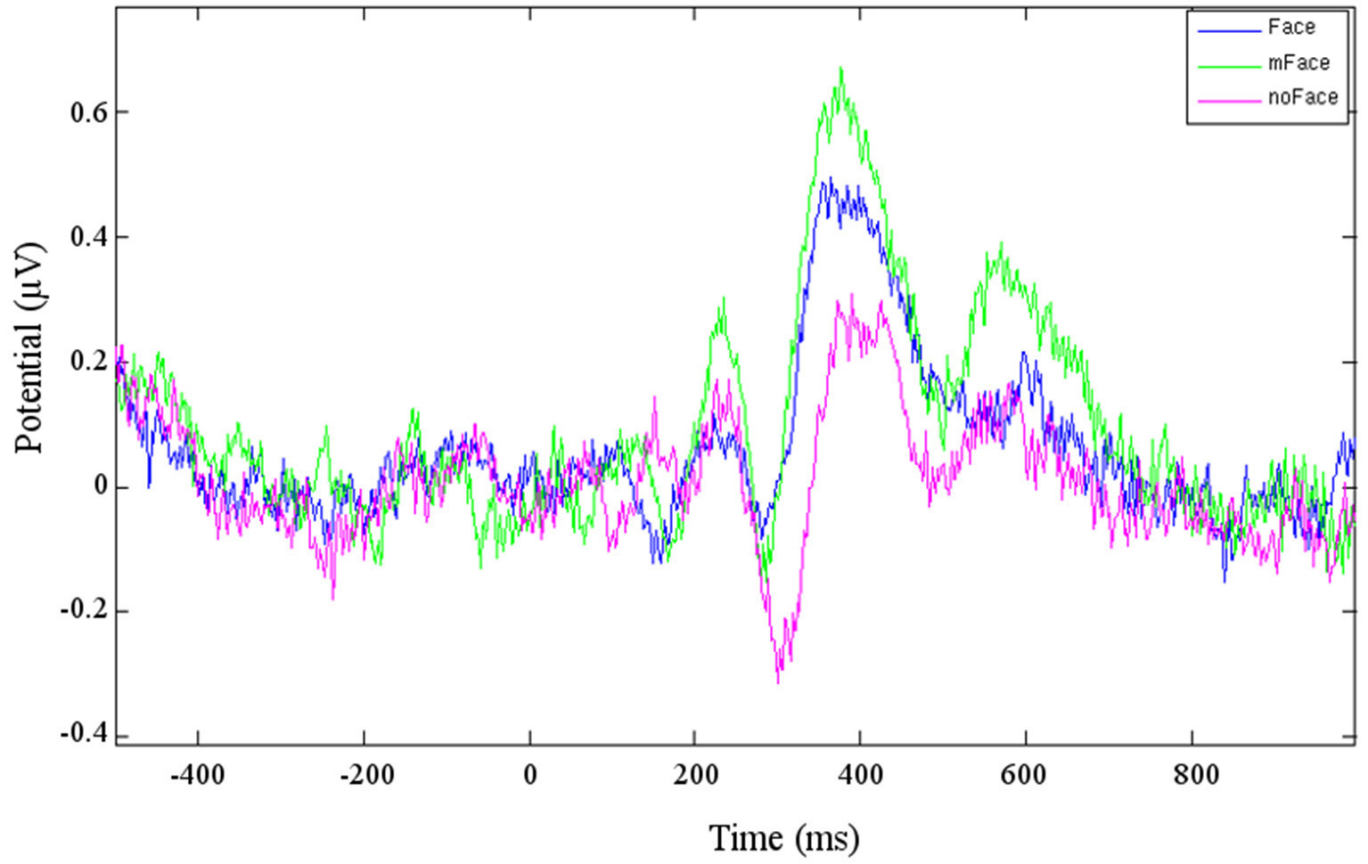
Averaged ERP of the three conditions of cluster 4.

Cluster 4 contains components from eight participants, the resulting ERP is shown in Figure 3. The negative peak starting at *t* = 280 ms is considered being the anticipated N170. The potential for the stimulus “no face” is clearly stronger than for “face” or “maybe face.” The following P1 has a distinctive peak for “maybe face” and is the weakest for “no face.” Cluster 8 is likewise located in the temporal lobe, but on the right hemisphere. A difference is that the “maybe face” causes the strongest N170 as well as P1 and the “face” the weakest.

Cluster 10 shows activity close to the vertex, and the ERPs for the three stimuli are similar to one another. The high similarity between the ERPs leads to the conclusion that Cluster 10 does not contain any “face-” related ICs.

**Figure 4:**
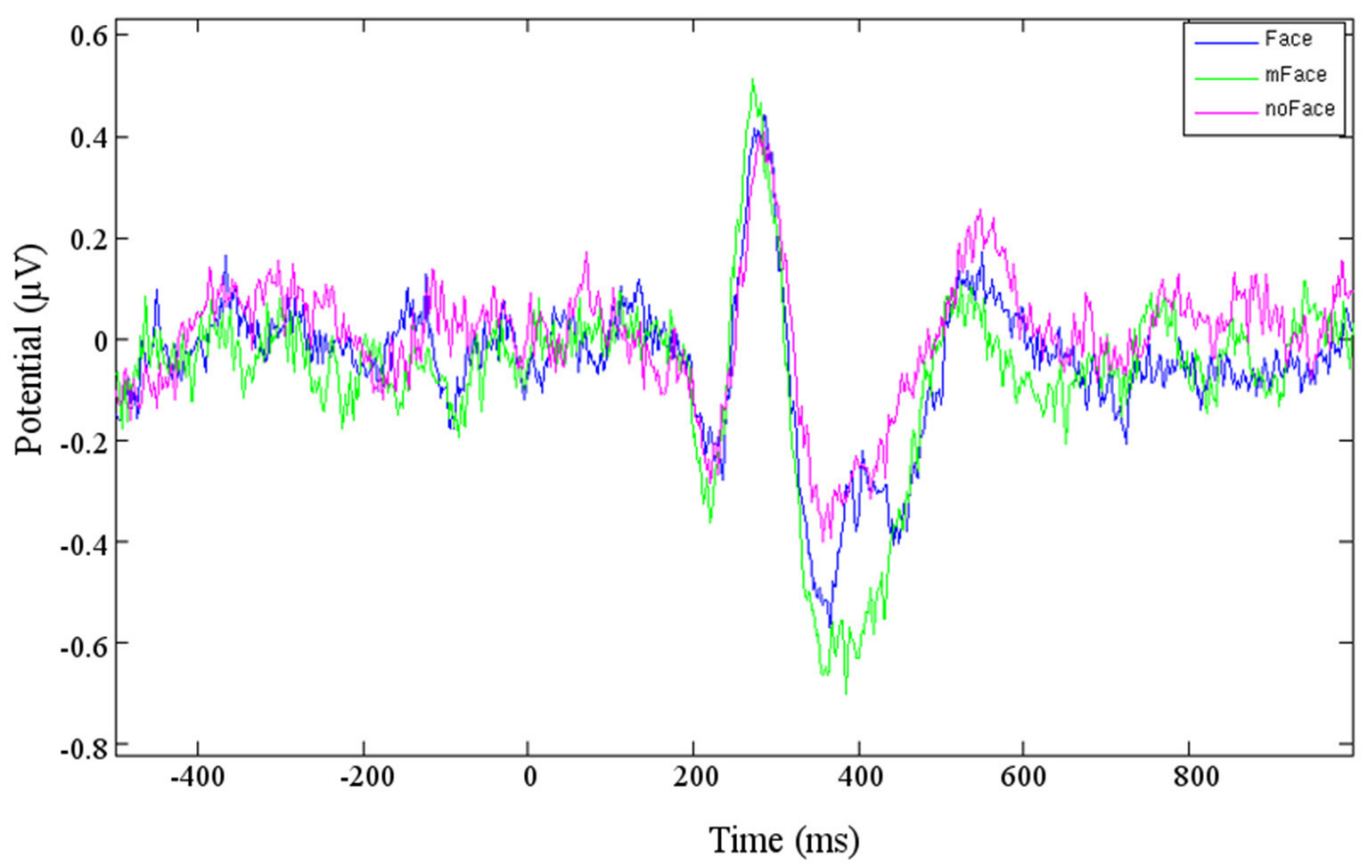
Averaged ERP of the three conditions of cluster 6.

Three clusters (3, 9, and 16) can be localized in the cingulate cortex, which can be divided into three bigger areas: anterior, posterior, and retrosplenial cingulate cortex. Cluster 3 and Cluster 9 contain together 10 different participants. The clusters are localized in the posterior cingulate cortex, while Cluster 16 is located in the anterior cingulate cortex. The ERPs of Cluster 6 show a pattern, apparently consisting of the N170 and P1 (Figure 4). For both ERPs, the strongest potential is evoked by “maybe face,” while “no face” is the weakest. Cluster 9 does not show the typical face-recognition characteristic. The N170 is weak for every type of stimulus, especially the potential caused by “face” is barely recognizable. Nonetheless, the P1 is of recognizable shape for all three types. For Cluster 16, the different stimuli do not evoke different potentials, and no characteristic pattern can be identified.

### Classification Results

In this section, we investigated the classification of data, containing only cortical components and in comparison to the data that include eye components. After identifying components displaying eye activity using the ICA, the classification could be run on data without the eye components or on solitary eye components. To improve clarity, “face” is abbreviated with F, “maybe face” with mF, “no face” with nF, and the merged dataset of “maybe face” and “no face” with mnF. Table 2 shows the grand-average classification results after removal of the eye components and when classifying only on the eye components.

**Table 2:** Misclassification results.

	**Marker**			
**Classification data**	**F vs. nF**	**F vs. mnF**	**mF vs. nF**	**F vs. mF**
Data after ICA and removal of eye components	27.75 (42.54)	26.61 (32.95)	35.12 (35.73)	27.48 (50.49)
Only eye components	39.31 (92.84)	37.90 (94.72)	46.27 (22.35)	37.09 (95.97)

*Average misclassification accuracy (variance) (%)*.

The grand-averaged misclassification rate for discrimination on the eye components in mF vs. nF, however, is 46.27% (variance: 22.35 %), meaning solely eye components are barely discriminable. The misclassification rate for the eyes for F vs. nF, in contrast, is 39.31% (variance: 92.84%), and the rate for F vs. mF and F vs. mnF is in a similar range. The rates mF vs. nF indicate that, in this case, eye movements are not too dominant. Eye movement in the other cases is slightly better distinguishable. The patterns representing the eyes could build some kind of distinguishable structure when overlapping. This possibility could also be given in the other classification cases, even if eye movements do not interfere as much, and is still a reason to discard the eye components. The resulting misclassification of F vs. nF without the eyes is 27.75% (variance: 42.54%).

**Figure 5:**
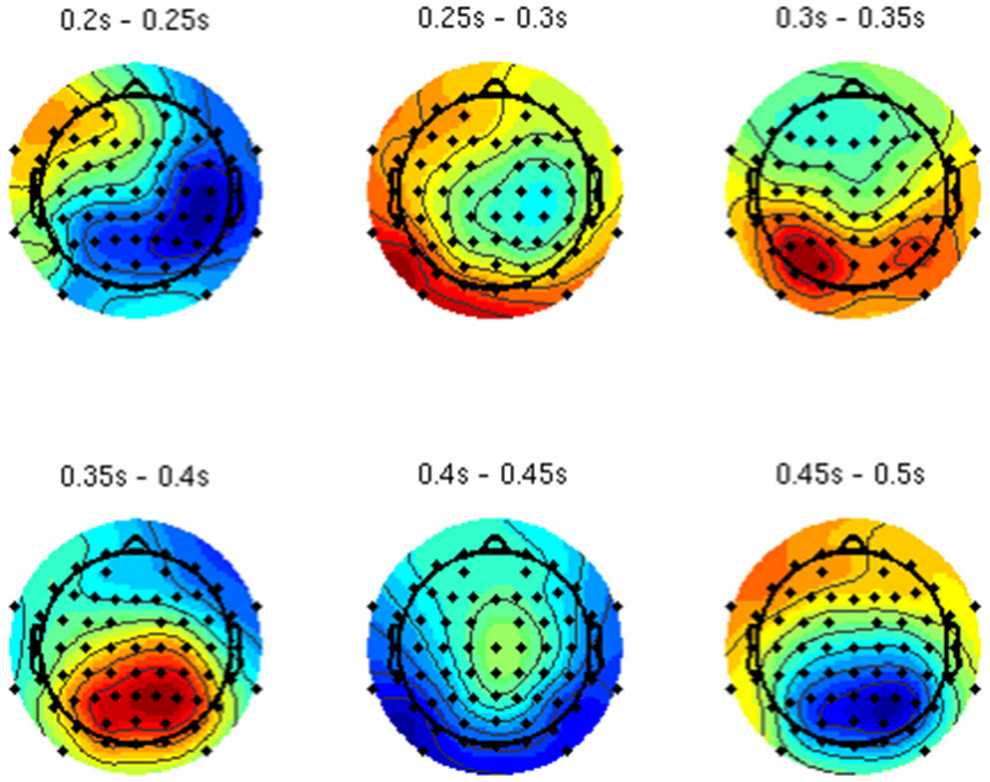
The pattern of ERPs (face vs. no face).

By analyzing the activation patterns resulting from the LDA in the Pattern Matching approach (Haufe et al., 2014; Krol et al., 2018), dipolar projections from cortical sources were identified (Figure 5). Specifically, the third time window (0.30–0.35 s) shows a clear projection pattern reassembling the projection from the fusiform gyrus, with stronger activity on the left hemisphere. This is in accordance with the assumptions from the literature on face recognition and fusiform activation.

### *Post-hoc* Analysis

In order to make an objective statement about the difference between the ERPs, a *t*-test with two paired samples was calculated for the ERPs of Clusters 6 and 4. First, all the average values along with the variances for the “face” and “no face” ERPs in Cluster 6 were calculated for the area of 286 ± 10 ms (time of the appearance of the N170). It has to be checked whether those values follow a Gaussian distribution before computing the *t*-test. Since the sampling size is rather small, this is done by using the Kolmogorov-Smirnov-Test. All three calculated cases follow a Gaussian distribution. Calculating the *t*-test for the N170 and P1 (*t* = 258 ± 10 ms) led to the result that the difference between the samples within Cluster 6 with *p* = 0.10 and *p* = 0.17, respectively, was not significant. Since Figure 3 already showed that the peak for “no face” is more negative than the one for “face,” the N170 of Cluster 4 did not need to be calculated. The average values of the P1 were calculated for a time range of ± 25 ms around 380 ms. The difference resulted in *p* = 0.60; therefore, the seen difference does not get significant either. Since the sampling size was rather small, this was done by using the Kolmogorov-Smirnov-Test; all calculated cases follow a Gaussian distribution.

### Restrictions of the Given Experimental Paradigm

Task-induced artifacts can reduce the reliability of apBCI approach as the noise level and likelihood for misclassification are increased. Possible artifactual influences resulting from the chosen experimental paradigm are discussed in this section. The first artifact coming to mind in the presented experimental design is context-related eye blinks. Eye blinks go along with task accomplishment and could, therefore, increase the noise. When concentrating on a task, especially on one which demands visual attention, eye blinks are suppressed. The suppression allows maintaining a stable visual perception as well as awareness. This causes eye blinks to occur immediately before and after the task (Nakano et al., 2009). In a study from Fukuda (1994), this could also be observed within visual discriminative tasks. Eye blinks were not inhibited during or before tasks, but after tasks completion frequent blinks could be recorded. The blinks concentrated between 370 and 570 ms after the stimulus onset, which indicates that they should not have an impact on the ERPs of interest. What occurs within that time frame though is incomplete blinks: The upper lid does not touch the lower one, but the relative lid's distance is reduced. This could have had an impact on the recorded EEG data and should be considered in further analyses. Secondly, as gating can be influenced by the fix inter-stimulus interval, some kind of anticipation artifact could occur. Low-frequency oscillatory activity is a mechanism that has been proposed to reflect gating (Buchholz et al., 2014). One characteristic of visual activity in anticipation of a visual event is a posterior oscillation from about 8–12 Hz, called the posterior alpha rhythm. It originates in the occipital-parietal area and arises during a wakeful rest (Romei et al., 2010). It is, furthermore, top-down controlled, also in discrimination tasks (Haegens et al., 2011). A negative relationship between the alpha power and perception/detection performance has been observed (Ergenoglu et al., 2004). Not only alpha, but also beta band oscillations correlated with sensory anticipation and motor preparation (Buchholz et al., 2014). The detection performance is, furthermore, influenced by oscillation bands (Ergenoglu et al., 2004; Hanslmayr et al., 2007). Therefore, it is likely that data recorded for this study are contaminated with oscillation caused by non-intended artifactual activity. The third interfering factor is the Readiness Potential (RP) due to a fixed ISI. It precedes self-paced movements and induces a slow negative shift. It can start up to 850 ms prior to the event and is bilaterally symmetrical (Libet et al., 1983). About 400–500 ms before the movement begins, the RP becomes asymmetric and larger over the contralateral hemisphere (Shibasaki et al., 1980). While it is positive in the frontal area, the RP has a negative maximum at the vertex. In the occipital area, it is recorded either absent or dismissable small (Deecke et al., 1969).

Another possible experimental influence to be pointed out here is the participant's response with the index finger. Within the paradigm, the participants had to indicate whether they saw a face or not. This should assure the participant's throughout attention but, at the same time, caused several measurable ERPs on the motor cortex and in areas preparing the movement. A high number of publications can be found investigating movement-related potentials. A study from Shibasaki et al. distinguishes between eight different components, pre-motion as well as post-motion (Shibasaki et al., 1980). Naming and allocation the potentials are inconsistent within this field of research. Only the two most considered potentials (beside the RP) regarding the movement will be mentioned in the following: the pre-motion positivity and the motor potential. A pre-motion positivity can precede the motoric response. It is about 100 ms before the movement onset (Böcker et al., 1994) and is diffusely spread over the scull and has high inter-individual variation (Deecke et al., 1969). Due to these features, it is not feasible to take the pre-motion positivity into account when making the analysis. Another possibly relevant factor is the motor potential, beginning about 60 ms before the movement. The motor potential has a maximum over the contralateral area, representing hand movement (Deecke et al., 1969). Deecke et al. consider the motor potential to be different from the RP for two reasons: First, the considered ERPs show an abrupt deflection before the onset of movement. Second, the distribution over the skull is different; the RP is mainly symmetrical while the motor potential is asymmetrical. Richter et al. did an fMRI study with a finger movement task (Richter et al., 1997). According to their results, pre-motor cortex and supplementary motor area (both BA 6, Grahn, 2013) show activity during movement preparation of the self-initiated movement as well as during execution. The primary motor cortex shows comparably weaker activity during preparation but is also very active when the movement is executed (BA 4, Grahn, 2013). Böcker et al. (1994) aimed at the activity before left or right hand movement. The readiness potential (about 900 ms before stimulus), the pre-motion positivity (about 100 ms before stimulus), and the motor potential (about 25 ms before stimulus) are close to the vertex of the brain.

## Discussion

The results presented here show that it, indeed, is possible to detect correlates of face recognition in single-trial EEG with a pBCI. Cluster 6 could be located in BA 18, which is the corresponding Brodmann area for the electrodes for O1 and O2. These electrodes are close to the electrodes PO9 and PO10 on which Deffke et al. (2007) focused in their study as well. The stimuli evoke an ERP in this area (Figure 4), which is very similar to the evoked potentials by Deffke et al. with the difference of a weaker amplitude. It is concluded that Cluster 6 probably represents the activity evoked when a face is recognized. Clusters 4 and 8 are localized in the temporal lobe. It was mentioned that facial response could also be observed in the temporal lobe. Furthermore, the shown ERPs are very similar to the face-related patterns described in literature. Both clusters are, therefore, recognized as indicators for face recognition as well. Clusters 3 and 9 both have their mean dipoles localized in the posterior cingulate cortex. That mainly correlates with retrieval of memory. This can be explained by the activation of the posterior cingulate cortex in the recognition of objects, places, or houses (Kozlovskiy et al., 2012). The localization of the mean dipoles in the occipital-parietal area (Clusters 6 and 8) and in the frontal area (Cluster 4) goes along with findings in the literature. All of these areas have also been relevant in the study from Bötzel et al. (1995). The ERPs are also very similar to the expectations resulting from literature review and have frequency components in the alpha band. In sum, these are very strong indicators that the experimental design did generate facial response in the brain, which is represented by those clusters.

After removal of the identified eye components, clear ERPs in the occipital-parietal area and close to the vertex were found. This is consistent with findings in the literature. On single channels, oscillations above 10 Hz were found and could represent alpha waves resulting from the Readiness Potential. Through band-pass filtering from 7 to 13 Hz, covering the interindividual differences in alpha would have ensured that the RP component is filtered out for each participant. Nevertheless, such strict filtering would have significantly affected the ERPs, diminishing the already small differences found between classes. One difference between the obtained and expected ERPs is an additional, short positive peak just before the main negative peak (~420 ms) that can be seen on PO9 and PO10 but not on Cz. Before that early potential, “face” is stronger negative than “no face,” which was expected. But later, “no face” is even more negative than “face.” Furthermore, the positivity evoked at roughly *t* = 600 ms at PO9 and PO10 could be the P1. In this case, the N170 and P1 would occur far apart in time. Comparing to the P1, “face” is stronger than “no face” though, which was also described in literature.

In all three cases, for F vs. nF, F vs. mnF, and F vs. mF, the grand misclassification rates for the eyes are about 38%. The variance is roughly 94%, which means that high variabilities existed between the participants. Considering the case mF vs. nF, the misclassification rate is 46.27%, variance: 22.35%. In this case, where no face stimuli were considered, the eye movements were less distinguishable. This leads to the assumption that, in all cases, eyes barely had any impact on classification. When face stimuli appeared, eye blinks locked to the stimuli could occur, causing a higher detection rate.

Classification rates without eye components are discussed in the following paragraph. The change in classification performance on raw data (misclassification rate: 25.33%, variance: 33.86%) to data after ICA cleaning (misclassification rate: 27.75%; variance, 42.54%) is minimal. Some clusters show a strong contamination with artifacts, as discussed in the previous section. Therefore, the clusters were removed, but the classification results did not improve much (misclassification rate: 28.91%, variance: 53.49%), leading to the conclusion that those artifacts did do not have significant impact on classification and can thus be ignored.

The pattern in Figure 5 shows a bi-dipolar activity in the occipital-parietal area, which was expected. It is an indicator that classification rates are based on features generated by the “face” stimuli. It is, furthermore, an indicator for a successful ICA: The solution allowed to identify the certain components (in this case, artifacts) and remove them, resulting in a pattern close to those expected by literature.

In order to apply the research idea of facial recognition to the automobile industry, a real-time detection of the N170 and P1 needs to be established with apBCI reliably and practically. This work showed that discrimination is, indeed, possible in a laboratory setup. A redesign of the experimental paradigm appears not to be relevant, as the artifacts that were identified here do not have a significant impact on classification performance. With that, the given calibration paradigm can be considered well working. The next step has to focus on the application in more realistic scenarios and in an online fashion. These findings could be extended using the results by Shishkin et al. (2016), who introduced a marker to differentiate between intentional and spontaneous eye movements. In addition, a real-world application of such a pBCI approach would need a sufficiently usable sensor system available. Research investigating such systems shows different approaches that might lead to ubiquitous solutions supporting the intended in-car application (Zander et al., 2017; Kosmyna and Maes, 2019; Vourvopoulos et al., 2019; Hölle et al., 2021). The recognition of a face combined with the intentional gaze would be a good indication of the intent to interact. The classification results of facial recognition would contribute to the detected intention to communicate, allowing the robustness of the system to increase.

## Conclusion

The expectation of finding sources for facial recognition in the occipital-parietal, the temporal area, and close to the vertex was met by the results presented. Two clusters show activity in the occipital-parietal area (Clusters 6 and 9). Furthermore, the mean dipoles of Clusters 4 and 8 are located in the temporal lobe. The ERPs of the Clusters 4, 6, and 9 show a clear N170 and P1; they are alike to those known from other face-recognition studies. The ERPs of Cluster 9 show slight differences, especially the N170.

The misclassification rates, displayed in Table 2, have a mean value of about 27.69%. These rates do not suffice for real-life application, especially if security issues are involved. Nevertheless, each correct classification can contribute to safety in autonomous driving. Thus, further research is indicated and improvements will bring this approach closer to real-life applications. In any case, these results do show that the patterns have a discriminable difference. The cluster study showed that the N170 and the P1 depicted by Cluster 6 were relatively strong in the fusiform face area. Cluster 6 could be located in the occipital lobe, BA 18. This is exactly the area describing the fusiform face area—this was expected in theory.

Due to the high variance of results between the participants, we suggest to include more participants in future studies. Specifically, before this very first approach can be taken further into the application domain, the overall classification accuracy needs to be improved, and it has to be investigated how far artifacts and loss-over-context control in realistic scenarios impact the classification. Also, the use of current EEG caps and electrodes hinders the real-world application. Further developments are needed in the engineering of EEG systems and in the usability of a pBCI with non-experts. With this study, the first step toward an automated detection of face recognition from EEG data is taken. With this, the idea of using the human brain as a sensor to support automated decision-making comes closer to reality and stimulates future research to identify other cognitive processes to be detected by a pBCI and be used in meaningful real-world scenarios.

## Data Availability Statement

The original contributions presented in the study are included in the article/supplementary material, further inquiries can be directed to the corresponding author/s.

## Ethics Statement

The studies involving human participants were reviewed and approved by Technische Universität Berlin. The patients/participants provided their written informed consent to participate in this study.

## Author Contributions

RPX and TZ: conceptualization, validation, and investigation. LA, RPX, and TZ: methodology. RPX: software, formal analysis, data curation, writing—original draft preparation, and visualization. TZ: resources, writing—review and editing, supervision, and project administration. All the authors have read and agreed to the published version of the manuscript.

## Conflict of Interest

By the time of paper writing, RPX was employed by the Volkswagen AG. The remaining authors declare that the research was conducted in the absence of any commercial or financial relationships that could be construed as a potential conflict of interest.

## Publisher's Note

All claims expressed in this article are solely those of the authors and do not necessarily represent those of their affiliated organizations, or those of the publisher, the editors and the reviewers. Any product that may be evaluated in this article, or claim that may be made by its manufacturer, is not guaranteed or endorsed by the publisher.
